# Nature of Phthisis, 235

**Published:** 1890-07-19

**Authors:** 


### NATURE OF PHTHISIS.

Dr. William Pearse, senior physician to the Plymouth Public Dispensary, has recently published some "Notes on subjects and questions of Practice." Among them we find "points in phthisis," which are well worth attention. Dr. Pearse remarks that the most obvious and great fact of phthisis is, that it happens mostly in the two decades—twenty-five to thirty-five, and thirty-five to forty-five. Another very striking fact is, that in a person who comes for some other complaint, phthisis may often be predicated from the physical aspect, or from some peculiar physical character of one or another part of the body. Conditions of hair, nails, teeth, and functional symptoms such as indigestion, amenorrhœa, lessened heat-production, no appetite, sense of sinking at the epigastrium, extreme sense of coldness of the feet at night, correlate with future phthisis. Long before any physical signs can be detected, a patient's main complaint is often of weakness, and especially morning weakness. All this Dr. Pearse considers points to an exhausted vital energy of protoplasm as more paramount and fundamental in the disease than is the subsequent development of bacilli in the living tissue. Slow evolution of phthisis is very common, extending not only over months, but over years. For instance, hæmoptysis may occur and recur over a series of years in phthisically-typed girls, and yet a most careful examination of the lungs fails to detect any phthisical signs. Accepting the bacilli hypothesis as part of the full phenomena of phthisis, the protoplasm yet retains sufficient energy for the evolution of normal cells, until some shock, *e.g.*, the exhaustion of child-bearing, or too long suckling, or the diminution of vital energy which age compels, is added to the previous condition. Dr. Pearse considers that if we look mainly to the lungs, we take a partial view of phthisis. The final lung disease is a minor part of the full disease.

